# Supplementary material for: Bid maintains mitochondrial cristae structure and function and protects against cardiac disease in an integrative genomics study
Source: eLife. 2018 Oct 3;7:e40907. doi: 10.7554/eLife.40907 (PMC6234033; doi:10.7554/eLife.40907)
Supplement: Transparent reporting form [file elife-40907-transrepform.docx]

***eLife’s* transparent reporting form**

We encourage authors to provide detailed information *within their submission* to facilitate the interpretation and replication of experiments. Authors can upload supporting documentation to indicate the use of appropriate reporting guidelines for health-related research (see [EQUATOR Network](http://www.equator-network.org/%20)), life science research (see the [BioSharing Information Resource](https://biosharing.org/)), or the [ARRIVE guidelines](http://www.plosbiology.org/article/info:doi/10.1371/journal.pbio.1000412) for reporting work involving animal research. Where applicable, authors should refer to any relevant reporting standards documents in this form.

If you have any questions, please consult our Journal Policies and/or contact us: [editorial@elifesciences.org](mailto:editorial@elifesciences.org).

**Sample-size estimation**

- You should state whether an appropriate sample size was computed when the study was being designed
- You should state the statistical method of sample size computation and any required assumptions
- If no explicit power analysis was used, you should describe how you decided what sample (replicate) size (number) to use

Please outline where this information can be found within the submission (e.g., sections or figure legend), or explain why this information doesn’t apply to your submission:

**Mouse experiments**

Sample size for echocardiogram analysis (Figure 4 and Figure 4 -figure supplement 3) was done using a power calculation as outlined in the Methods section on page 26. All mouse echocardiogram results are also discussed on pages 6-7 of the Results section.

**Tissue culture**

All cell culture experiments in the main figures were biologically repeated a minimum of 3 times, the majority of which were repeated 4 times or more, as indicated in legends.

**Human genetics**

The increased power of the PrediXcan (Gamazon et al, Nature Genetics, 2015) or TWAS approach (Gusev et al, Nature Genetics, 2016) was explored in the original publications. Indeed, there was significantly improved power relative to the approach using the top eQTLs (which also had already significantly greater power than traditional SNP-based approach).

**Replicates**

- You should report how often each experiment was performed
- You should include a definition of biological versus technical replication
- The data obtained should be provided and sufficient information should be provided to indicate the number of independent biological and/or technical replicates
- If you encountered any outliers, you should describe how these were handled
- Criteria for exclusion/inclusion of data should be clearly stated
- High-throughput sequence data should be uploaded before submission, with a private link for reviewers provided (these are available from both GEO and ArrayExpress)

1. For all experiments and human genetics data, information concerning the number of replicates is provided in each figure legend (noted as n=) or within the Results section of the corresponding section (for example the number of individuals in a GWAS cohort).

2. Biological replicates are defined in the statistics portion of the Methods section and are as follows: the same experimental method independently tested on different samples of the same type of cell or mouse model.

3. For human genetics studies, we replicate all findings in independent cohorts as stated throughout the results section. Please see the above comments for number of patients.

4. For epinephrine treated mice presented in Figure 4c-g, one *Bid-/-* was not included as it was a clear biological and statistical outlier (based on Grubbs’ outlier test, p<0.05). This is noted in the statistics Methods on page 27.

5. GTEx v6 data (high throughput transcriptome sequence data) have been made available to the scientific community from the GTEx portal (<http://www.gtexportal.org>) and dbGap (GTEx Consortium, Nature, 2017).

Please outline where this information can be found within the submission (e.g., sections or figure legends), or explain why this information doesn’t apply to your submission:

**Statistical reporting**

- Statistical analysis methods should be described and justified
- Raw data should be presented in figures whenever informative to do so (typically when N per group is less than 10)
- For each experiment, you should identify the statistical tests used, exact values of N, definitions of center, methods of multiple test correction, and dispersion and precision measures (e.g., mean, median, SD, SEM, confidence intervals; and, for the major substantive results, a measure of effect size (e.g., Pearson's r, Cohen's d)
- Report exact p-values wherever possible alongside the summary statistics and 95% confidence intervals. These should be reported for all key questions and not only when the p-value is less than 0.05.

Please outline where this information can be found within the submission (e.g., sections or figure legends), or explain why this information doesn’t apply to your submission:

1. A comprehensive description of all statistics are provided in the Methods section of the paper, as well as in detail for each figure panel within the legends (when applicable).

2. Raw data has been provided throughout the manuscript and in figures: 1.) in the form of individual data points when biologically pertinent (see: Figure 4c-d and Figure 4-figure supplement 3, Figure 6-figure supplement 4e, and Figure 6b) and 2.) for human genetics analysis (Figure 7b and Figure 7-figure supplemental 6c).

3. Exact statistical tests, N values, and dispersion (means) and precision measurements (SEM and 95% confidence intervals) are reported for each panel in figure legends (and elaborated upon with the Results) where applicable.

3. Exact p-values are reported when biologically important comparisons are shown within figure panels and the Results section of the manuscript (See Figures 6b, 7b, 9c and Figure 4-figure supplement 3a, Figure 6-figure supplement 5e, Figure 7-figure supplement 6c, Figure 7-figure supplement 7c).

(For large datasets, or papers with a very large number of statistical tests, you may upload a single table file with tests, Ns, etc., with reference to sections in the manuscript.)

**Group allocation**

- Indicate how samples were allocated into experimental groups (in the case of clinical studies, please specify allocation to treatment method); if randomization was used, please also state if restricted randomization was applied
- Indicate if masking was used during group allocation, data collection and/or data analysis

1. Indicate how samples were allocated into experimental groups:

Our study is a case control study that compares groups (disease or not), which are defined by electronic health records data, retrospectively.

2. Indicate if masking was used: Masking was not used.

Please outline where this information can be found within the submission (e.g., sections or figure legends), or explain why this information doesn’t apply to your submission:

**Additional data files (“source data”)**

- We encourage you to upload relevant additional data files, such as numerical data that are represented as a graph in a figure, or as a summary table
- Where provided, these should be in the most useful format, and they can be uploaded as “Source data” files linked to a main figure or table
- Include model definition files including the full list of parameters used
- Include code used for data analysis (e.g., R, MatLab)
- Avoid stating that data files are “available upon request”

1. Relevant data files: We use publicly available GWAS data and electronic health records data (e.g., UK Biobank), which are public or available through an application process, and publicly available transcriptome/genotype data from the GTEx Consortium (v6p), which are available through the GTEx portal (<http://www.gtexportal.org>) and through dbGap (GTEx Consortium, Nature, 2017).
2. Include model definition files : These are described in (Gamazon et al., *Nature Genetics* 47:1091 (2015) ).
3. Code for the following analyses is publicly available:

   PrediXcan: https://github.com/hakyimlab/PrediXcan
   S-PrediXcan: https://github.com/hakyimlab/MetaXcan
4. We have provided this information in the Data Availability section of the Methods.

Please indicate the figures or tables for which source data files have been provided:
